# Impact of Psychosocial Intervention on Performance Determinants in Competitive Swimmers: Roles of Coach, Family, Environment, and Athlete Characteristics

**DOI:** 10.3390/sports13090314

**Published:** 2025-09-09

**Authors:** Alejandro López-Hernández, Juan Ángel Simón-Piqueras, David Zamorano-García, David B. Pyne, José María González Ravé

**Affiliations:** 1 Faculty of Sports Sciences of Toledo, University of Castilla-La Mancha, 45004 Toledo, Spain; alejandro.lopez@uclm.es (A.L.-H.); josemaria.gonzalez@uclm.es (J.M.G.R.); 2 Faculty of Education of Albacete, University of Castilla-La Mancha, 02017 Albacete, Spain; 3 Research Institute for Sport & Exercise, University of Canberra, Canberra 2617, Australia; david.pyne@canberra.edu.au

**Keywords:** parents, family, expert performance, swimming, sports environment, coach characteristics

## Abstract

(1) Background: This study analyzed the effects of intervention programs conducted between 2021 and 2025 as part of the “Aula UCLM-FNCLM” initiative—a partnership between a regional swimming federation and the university—on psychosocial factors in trained swimmers. This program presents interventions for swimmers, their coaches, their families, and their sports environment. (2) Methods: The effects of a 4-year intervention program on the Castilla-La Mancha swimming team, classified as Tier 3 (Tier 3: Highly Trained/National Level), according to the McKay classification framework (2021 team with 55 swimmers, 25 men, and 30 women; 17.3 ± 5.3 years), and the same sample in 2025 (42 swimmers, 19 men, 23 women—17 ± 2.8 years—and 11 dropouts). A self-perception questionnaire on performance in sport (CAED) was used. (3) Results: The results showed higher ratings (*p* < 0.001, *η^2^* = 0.30) from the swimmers of the 2025 CLM team in the coach factor, and moderate changes in the roles played by family (*p* < 0.05, *η*^2^ = 0.12) and environment (*p* < 0.05, *η*^2^ = 0.11). The only factor that did not improve was personal characteristics. (4) Conclusions: The UCLM-FNCLM program has contributed to improving the role of factors related to performance in sport (coach, family, and sports environment) in a cohort of regional-based swimmers.

## 1. Introduction

Studies on expert performance in sport have evolved from prioritizing deliberate practice [1] to models that include other types of psychosocial and organizational factors relevant to achieving that performance [2]. Several studies have questioned the number of working hours and the variability of the impact of this theory [3,4,5]. Identifying what constitutes deliberate practice depends on a combination of factors related to personal aspects, the role of the coach or guide, and the organizational environment. To promote expert performance, it is essential to integrate these elements in a coherent manner adapted to the specific learning context [6]. A six-factor model related to expert performance was established in Spanish athletes, two of which relate to deliberate practice, while the others relate to various types of factors [7]. Analysis of this approach indicated that personal characteristics were the most important factor perceived by athletes, above factors related to training. Of the other factors, the role of the coach was more important than family and, in turn, more important than environment [7].

One of these factors addresses the coach’s characteristics detailing items referring to knowledge, treatment, emotional involvement, and involvement in the athletes’ personal lives. The quality of the relationship between coach and athlete is clearly fundamental. The 3+1 Cs model comprising closeness, commitment, complementarity, and co-orientation is essential for the performance of high-performance athletes [8]. Thus, the coach’s behavior and relationship with the athlete correlate positively with athletic outcomes [9]. Furthermore, coaches who can foster a positive motivational climate are crucial for athletes’ athletic commitment. Coaching styles that support athletes’ needs can reduce demotivation and increase autonomous motivation [10]. On the other hand, regarding their personal involvement, an element highly valued by athletes is related to the coach’s concern for the care and health (in the broadest sense of the term) of their athletes [11].

Another important factor in expert performance is of course an athlete’s personal characteristics, where high-level athletes possess greater skill in the emotional, psychological, physical, and cognitive aspects of sports. Competencies underlying sporting performance include self-confidence, motivation, commitment, attention regulation, arousal regulation, planning, evaluation, emotional regulation, stress management, resilience, mental toughness, coping, goal setting, imagery, and self-talk [2]. The personal characteristics of elite athletes are multifaceted and encompass motivation, personality, psychological well-being, stress management, and sleep quality. Understanding these characteristics can help optimize elite athletes’ performance and well-being [12,13].

The family factor reflects the high level of parental involvement in all aspects of their children’s athletic careers, providing significant emotional support and making substantial personal and financial sacrifices [11]. These aspects are crucial and multifaceted, influencing both young people’s participation in and enjoyment of sport. This high level of involvement may or may not be desired by athletes. Athletes prefer parents to show interest by facilitating and prioritizing their participation, listening and learning from them, and supporting them before, during, and after sporting events [14]. Although young people value support in terms of health and social development, and the fostering of self-determined motivations, they often consider parental intervention in aspects of sport and relationships with teammates and opponents undesirable. Excessive parental interference can negatively affect the development of children’s identity and autonomy [15,16,17].

The fourth factor is related to the sports environment and the type of resources available in the environment to provide emotional support and confidence to the athlete in order to promote their development. A case study conducted by Woods et al. [18] indicated that high-level sports organizations have used ecological dynamics frameworks for the preparation of performance of athletes, yielding excellent results, based on the integration of talent development with specific sports ecosystems. Another model illustrated athletic talent development environments using a holistic ecological approach highlighting the role of inter-organizational collaboration in talent development [19]. The quality of sports talent environments in five European countries was evaluated by comparing the perceptions of athletes, parents, and coaches [20]. The general consensus is that the ecological dynamics framework suggests that performance preparation should focus on interactions between the athlete and their environment, promoting more adaptive and contextualized learning [18,19,20]. This process can play a significant role in self-regulation and athletic performance. Athletes can use their social environment to set goals, select strategies, and manage their emotions during competition [20].

While all the factors in the model are relevant, it is worth analyzing whether one or more of them play a positive role adequately [7], as well as longitudinally over the important development years in a young athlete’s career. Intervention programs can improve expert performance in sport, with the majority conducted under a unifactorial approach [21,22]. It seems plausible that a multifactorial approach could yield more positive results. These approaches, based on dynamic systems theory, indicate that expert performance arises from the interaction of multiple personal and environmental constraints, so programs must be flexible and adaptive [23,24].

A review of the swimming literature does not show studies investigating longitudinal psychosocial programs over several years in highly trained subjects. The programs found under the label of psychosocial focus on psychological development to improve swimming performance over a few months [25,26] or isolate other types of factors (coach, parents) [27,28] or perspectives (holistic, positivist or pedagogical, etc.) [26], typically ignore others factors and their interaction in the development of athletes. In addition, in studies focused on swimmers, the level of training of the swimmers is not clearly reported. Based on these dynamic approaches, in 2022, the Castilla-La Mancha Swimming Federation (FNCLM), together with the University of Castilla-La Mancha (UCLM), launched a psychosocial intervention program called UCLM-FNCLM Classroom to promote swimming and health. This swimming initiative was established at the university to enhance performance in swimming, and it included intervention workshops, parental conferences, and psychological training programs designed to enhance performance-related factors. This program was designed specifically for this purpose and has not been used previously.

The aim of this field-based study was to evaluate whether the intervention programs implemented between 2022 and 2025 under the UCLM-FNCLM Classroom initiative positively influenced swimmers’ perceptions on critical factors associated with athletic performance. The hypothesis of this work was that application of this program would improve the perception of the swimmers participating in the study about the role that their personal characteristics, their family, their coach, and their sports environment have on their sports results.

## 2. Materials and Methods

### 2.1. Participants

The original sample in 2021 consisted of 55 swimmers. The mean age was 17.3 ± 5.3 years, with 25 men and 30 women. The mean number of years competing for the group was 8.6 ± 4.9, and the mean number of hours of weekly training was 9.8 ± 3.9. All swimmers were classified as Tier 3 (Tier 3: Highly Trained/National Level) according to the McKay classification framework [29]. At the end of the program, 13 swimmers had left the team for various reasons: low performance (4), injuries (1), change of region due to studies (5), and others (3). The mean age in 2025 is 17 years (SD = 2.75 years), with 19 men and 23 women. The mean number of years competing among the group was 9.18 (SD = 3.1 years). The mean number of hours of weekly training was 10.1 (SD = 2.5 h). The minimum number of participants required for the statistical analyses of the was ensured (Section 2.6). Swimmers who replaced swimmers who dropped out of the program during the program were not included in the study.

### 2.2. Design

This study employed a pre–post longitudinal design with empirical analysis. The study analyzed the influence of a program called UCLM-FNCLM classroom to promote swimming and healthy living, applied between 2022 and 2025 in swimmers from the Castilla-La Mancha region (Spanish region). The study was purely ecological, evaluating the Castilla-La Mancha regional swimming team attending national and international competitions. Establishing a control group of other regional swimmers was deemed inappropriate for many reasons, because they would not have had similar resources beyond the program itself, nor the same level of demands. A control group is both ethically unjustifiable and logistically unfeasible in this setting.

### 2.3. Questionnaire on Self-Perception of Performance in Sport

Four sub-scales of the instrument were administered: Coach, Sport Environment, Personal Factors, and Family. The factor of Gender and Age was used as a covariate. The questionnaire on self-perception of performance in sport (CAED) included four dimensions [7]. The dimensions were Coach, with 12 items, (example: your knowledge of the sport or event; your personal treatment, etc.); Sport Environment, with 10 items, (example: concern for my personal well-being; availability of material resources, etc.); Personal Factors, with 12 items (example: my ability to learn from my sport; my concentration in training); and Family, with eight items, (my family’s sacrifice for my sports career, as well as their emotional support). This questionnaire begins with the phrase “What I think is the reason for the improvement in my sport”. Items were scored on a 10-point Likert scale, ranging from “strongly disagree” (1) to “strongly agree” (10).

The Coach factor contains items that describe characteristics of coaches that facilitate athletic performance. These include elements such as the way they treat athletes, their involvement in both their athletic career and personal life, their knowledge of their sport, and their personal characteristics. The Sports Environment factor included items that provided information on need satisfaction, financial aid, material resources, and support staff, along with items related to the environment’s ability to meet athletes’ emotional needs. The Athlete factor explored certain personal and emotional characteristics of athletes. Finally, the Family factor contained items related to the different roles that athletes’ parents can play in fostering their athletic careers, as well as items about the parents’ personal and financial efforts.

Validation of the instrument in the Spanish population [7] yielded the following indices: *Reliability* = 0.949; *Cronbach’s alpha* = 0.80 in the lowest factor and = 0.94 in the highest factor: *CFI* = 0.87; *TLI* = 0.904; *GFI* = 0.923; and *RMSEA* = 0.048.

### 2.4. Procedure

Swimmers from the Castilla-La Mancha Swimming Federation (Spain) were contacted through the Federation’s staff. The participants provided their data through the questionnaires used in a completely anonymous manner, and this information was administered and analyzed only by the research team. The average time required to answer the questionnaire was approximately 15 min. All participants voluntarily took part in this study after having been duly informed in advance of all aspects of the study and having signed the pertinent informed consent forms, including parental approval for underage swimmers. Subsequently, once the information had been gathered, it was downloaded in .csv format and imported into a statistical software application for analysis. All the consent forms were stored in .pdf format.

### 2.5. Program

At the end of 2021, the Castilla-La Mancha Swimming Federation, in collaboration with the University of Castilla-La Mancha, identified the need to address athlete performance beyond purely technical aspects. As a result, the Aula UCLM-FNCLM program was launched in 2022 to promote the holistic development of swimmers. The program has been continuously implemented through 2025 and is structured around four main areas of action.

A comprehensive professional development plan was implemented to enhance the competencies of swimming coaches within the community, grounded in continuous training, systematic evaluation, and structured mentorship. The first year focused on diagnosing existing capabilities and delivering foundational instruction in technical and pedagogical principles. In the second year, efforts were directed toward professionalization through certification processes, expert-led seminars, and the integration of technological tools. The third year emphasized methodological innovation and the cultivation of leadership skills. In the fourth year, the progress achieved was consolidated through comprehensive evaluations, the standardization of best practices, and the strategic dissemination and projection of the work undertaken. The percentage of participation of the coaches selected for inclusion in the program was to 95–98%.

Recognizing the key role of the family environment, the “Families of Swimmers” program was developed with the aim of enhancing the emotional support provided by families to swimmers. To achieve this, various actions were implemented focusing on emotional regulation, assertive communication, and understanding the stages of athletic development. A specific initiative, the “Parent School,” was created as part of the program. During the first year, the focus was on developing parents’ emotional awareness, helping them identify and understand their own emotions within the sporting context and their impact on the athlete’s well-being. In the second year, the program addressed strategies for emotional regulation and assertive communication, aiming to improve the quality of dialog between parents and children and to foster a supportive, pressure-free environment. The third year concentrated on consolidating the role of parents as active agents of emotional support, promoting resilience in the face of setbacks, encouraging athlete autonomy, and fostering a family environment that values the developmental process over competitive outcomes. For this program, the face-to-face activities were complemented with various online resources that allowed for asynchronous participation, and the participation of the families of the athletes was controlled. The loyalty rate of the program was over 95%.

Over a four-year period, the program was implemented with the aim of strengthening the social climate surrounding athletes, with a particular focus on group cohesion, motivation, and a sense of belonging. In the initial phase, assessments of group climate were conducted, and integration activities were carried out to foster positive interpersonal connections. Subsequently, the program promoted the construction of a shared identity through collective symbols, the celebration of group achievements, and intergenerational activities. In the third year, autonomous motivation and peer support were encouraged through internal mentoring systems, self-awareness workshops, and reflective dialog sessions. Finally, in the fourth phase, these practices were consolidated as part of the organizational culture through coach training, the establishment of well-being protocols, and ongoing evaluation mechanisms. Again, the percentage of active participation in this program was close to 90–94%.

**Figure 1:**
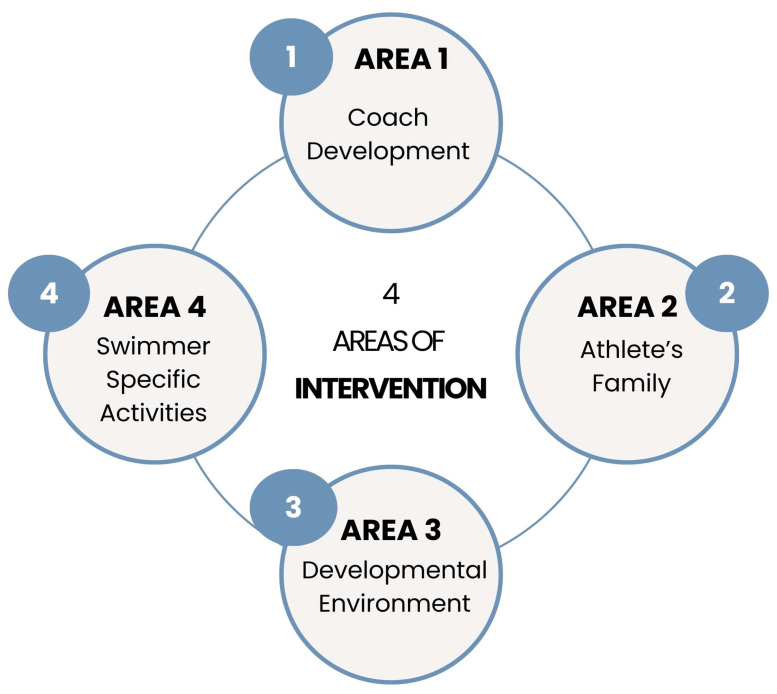
Four areas of the intervention within the Aula UCLM-FNCLM framework.

A new competitive program was implemented in which all athlete-directed interventions were tailored according to their competitive level and developmental stage. The objective was to provide meaningful experiences and ensure that all athletes felt valued within the program’s framework. Each year, the competition program evolved with an emphasis on athletes’ well-being, adequate rest, and excellence in competition. Indeed, the competition programs were differentiated based on the athletes’ age and level. In addition to competitions, related performance activities were conducted, including training camps, group reflections, and technical talks that supported personal, educational, and social development. Talent development programs, academic support, and dual-career pathways were key components. Regional teams were established—ranging from swimmers aged 13–14 years (male) and 11–12 years (female) to university level—as a means of athlete recognition, motivation, and monitoring (Figure 1). All swimmers participating in the study were part of this program.

### 2.6. Statistical Analysis

To calculate the minimum sample size required for analysis of the study, the number of participants was calculated using the G*Power 3.1 application. The expected effect size (f = 0.25), the associated probability of error (*p* = 0.05), and the desired statistical power (1 – *β* = 0.80) were considered, yielding a minimum sample of 36 participants. After data collection, we first calculated whether the sample followed a normal distribution. The tests showed that the sample followed a normal distribution in the variables used—Coach, Sport Environment, Personal Factors, and Family (1, 42) > 0.948, *p >* 0.05, using the Shapiro–Wilk test.

The Mauchly test of sphericity was significant in all the factors analyzed (*p* < 0.05), and therefore, Huynh–Feldt correction was used to analyze the significance of the intra-subject results. Using Levene’s test, the homogeneity of the sample was tested via the four factors—Coach (1, 40) = 0,49, *p* > 0.05, Sport Environment (1, 40) > 0.52, *p* > 0.05, Personal Factors (1, 40) = 0.32, *p* > 0.05, and Family (1, 40) = 0.35, *p* > 0.05. Box’s M test was carried out, obtaining significant results, and thus, in the rANOVA calculations, Pillai’s Trace was used. The software used for all statistical analyses was IBM SPSS 28.0 for Mac.

We ran a repeated measures analysis of variance (rANOVA), using, as repeated measures (independent variables), the pre–post data, as well as the four sub-scales selected of the CAED as dependent variables, and as a covariate, we used the gender and age of the participants. Experimental mortality was controlled in the statistical analysis with the list-based option. After performing the rANOVA, the estimated marginal means, and the upper and lower limits were calculated using the Bonferroni test. Finally, we calculated the effect size of each test and the power of the effects obtained (error α 0.05). The criteria established to determine the power of the effect in the post hoc results; the criteria were low (0.20), moderate (0.50), and large (0.80) [30].

## 3. Results

### Multivariate Results of the ANOVA

**Figure 2:**
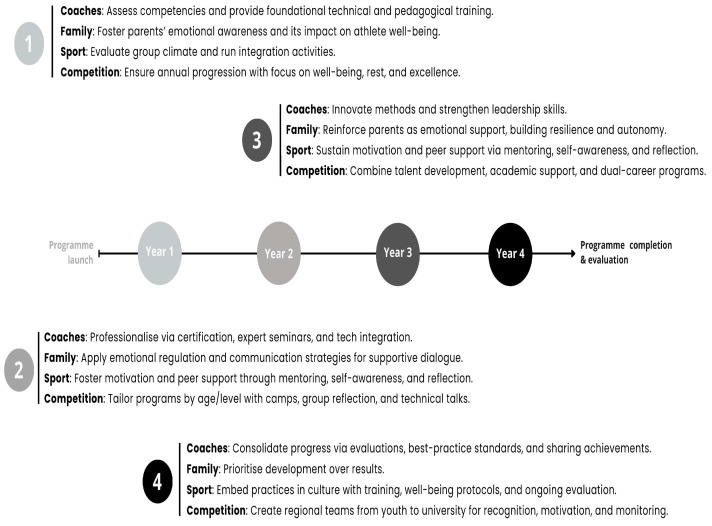
Timeline of the Aula UCLM-FNCLM framework.

**Figure 3:**
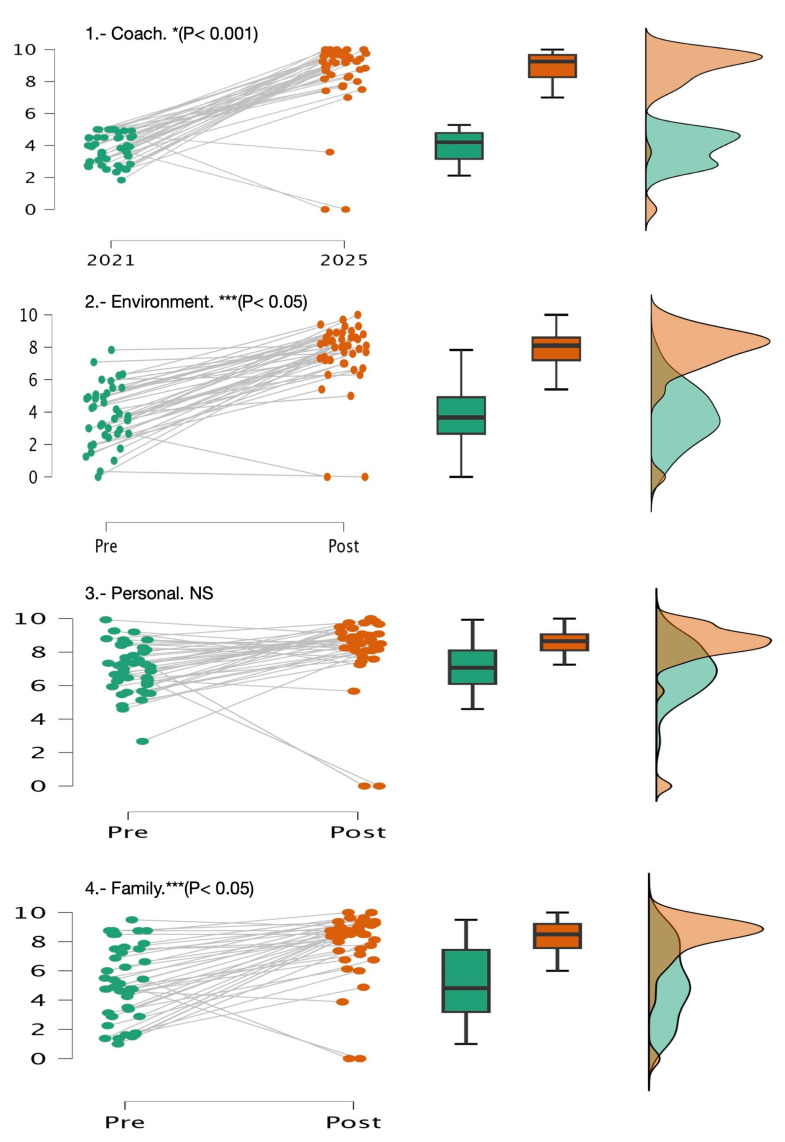
Changes in psychosocial domains. * (*p* < 0.001), and *** (*p* < 0.05). NS = non-significant differences.

The multivariate tests yielded a significantly higher score in Coach—*F*_(1)_ *=* 17.63, *p* < 0.001, *η*^2^ = 0.30, 1 − *β* = 0.98. Moreover, there were significant differences with medium effect sizes in Environment (*F*_(1)_ *=* 12.45, *p* < 0.05, *η*^2^ = 0.11, *1 − β* = 0.58) and Family (*F*_(1)_ *=* 12.58, *p* < 0.05, *η*^2^ = 0.12, 1 − *β* = 0.60) (Table 1 and Figure 2). No significant differences were found in Personal Factors and between any of the dependent and covariate variables (sex and age). Therefore, no influence was observed due to the gender and age of the participants *p* > (0.05) (Table 1 and Figure 3).

## 4. Discussion

Existing studies have not evaluated longitudinal psychosocial programs over several years in highly trained subjects. Therefore, to our knowledge, this is the first study of these characteristics in trained swimmers. The results showed that swimmers from the Castilla-La Mancha regional swimming team in 2025 have exhibited a substantial improvement in their perceptions of most factors related to athletic performance compared to their perceptions at the end of 2021. A significant improvement was observed in the role played by the coach, along with significant but moderate changes in the roles of family and environment, following the intervention programs implemented during 2022–2025. The only factor that did not show a significant improvement was related to personal characteristics. The hypothesis of this work was that application of this program would improve the perception of the swimmers participating in the study about the impacts that their personal characteristics, their family, their coach, and their sports environment have on their sports results. We have shown that the program has been very effective (based on the power of the effect obtained) regarding the Coach factor and effective at the medium level for the Environment and Family factors. On the other hand, the longitudinal intervention program was not effective in improving the perception of Personal Factors.

The multi-component program of activities was successful in improving the support of coaches, family, and environment. The inability of the intervention program to change the perception of personal factors could relate to a high baseline perception that the swimmers had prior to the program. Given the participating swimmers have a good level of competition and training performance when they started the program, there may have been a ceiling effect for this factor. The swimmers were highly trained in well-supported coaching programs and had achieved good success by selection in national and international junior squads and competitions.

### 4.1. Coach Development

The intervention programs were proven to be highly effective in enhancing the swimmer’s perceptions of the coaches’ competencies in teaching methodologies, performance analysis technologies, coaching, and leadership. Emphasis has been placed on the role that coaches can play, providing guidance on training aspects, as well as training on athletes’ psychosocial aspects, feedback, motivation, group management, emotions, etc. All these elements have been highlighted as relevant to the role of the coach in junior and senior sports. In the theory of deliberate practice itself, Erikson has already spoken of the fundamental role of coaches or guides in carrying out proper deliberate practice [6]. Cumming et al. [31] emphasized that some of the elements implemented in the evaluated program, including knowledge of the best coaches, created an environment conducive to performance, fostering skill acquisition, promoting enjoyment of sports activities, properly organizing the work group, and fostering the athletes’ psychosocial development. On the other hand, other investigators [32] have noted how coaches can, through the structure of the session and with their behavior in it, promote the learning of their athletes. Improvement in these elements was noted systematically during the period of implementation of the program.

The role of the coach and other trainers is clearly critical in high-performance sports. The key characteristics of efficient trainers in the current study [33] include a priority consideration. The coach’s communication skills (feedback), the confidence shown, and establishing positive relationships were priorities in the Aula UCLM-FNCLM program. The coaches understood that they are key elements in the psychosocial training of the swimmers, and according to the results, they appear to have modified their way of working with the athletes throughout the program. These results are in line with a transformational leadership perspective, through which developmental considerations and how they are adapted within the context of sport [34] were discussed. Other aspects, such as the feedback provided and the ability to create ideal working environments, are highly influenced by the coach’s knowledge and ability to transmit it to their athletes [35].

Other elements that were also positive in the activities of the coaches were ensuring a personal approach; respecting the athletes’ opinions; showing acceptance of, and support for, the athletes’ sporting decisions; and involvement in their personal lives. This assessment may reflect that these athletes’ coaches were able to maintain a higher-quality relationship with their athletes [8,9]. A coach’s ability to maintain a positive relationship with their athletes likely creates close bonds between them and maintains a more efficient coaching relationship. At the same time, the athletes perceived their coaches as individuals with a stable and trustworthy character [10]. The swimmers’ perception of their coaches in 2021 was quite low. However, the actions and follow-up provided by the AULA program seems to have had an impact on the coaches and influenced their way of working. Thus, the coaches were more highly valued by their own swimmers. It should be noted that, from 2021 to the present, no changes were made to the coaching staff of the swimmers included in the regional team.

### 4.2. Developmental Environment

The role of the intervention program in the Developmental Environment domain was to ensure a socially and structurally supportive environment for athletes. The results showed significant improvement in the perception of the sporting (swimming) environment with moderate effect power. This change could relate to initiatives in talent development, innovative competition plans tailored to the swimmers’ characteristics, and actions related to reconciling academic and athletic careers. The results are consistent with other studies analyzing the role of the sports environment in isolation. A focus on long-term development and a supportive social network is crucial for positive outcomes regarding talent development environmental characteristics (especially those regarding long-term development, effective communication, social networking, and psychological safety), being significantly associated with outcome variables [36]. Similarly, the importance of participating in special programs that include resources and support staff can be beneficial. These special programs are implemented within organized structures, which can range from schools to sports federations or clubs, depending on the country. The process of developing natural abilities is influenced, among other factors, by the environment in which the athlete operates, which includes the availability of programs and services, technical support, and support staff [37]. In our case, the program used in this study has a double function. The program was developed to directly influence the athletes involved in the program on the key environmental aspects. On the other hand, the program also fostered awareness of the importance of such factors for the athlete relative to other types of sports, as well as administrative and educational entities that generate a positive effect on other athletes.

No less important is the effect that satisfaction of emotional needs related to an athlete’s membership in a sporting program can have on performance. The perception of satisfaction of emotional needs appears to increase as the sporting level increases. Higher-level athletes feel more supported and better treated by sporting institutions. A high level of self-abstraction, which emphasizes social over personal identity, leads to more positive and less negative emotions at both the individual and group levels, which, in turn, improves team and individual performance. Social identity and its association with the team’s referent emotions is likely one of the key dimensions of emotion–performance relationships in team sports [38]. Emotional satisfaction in the social environment plays a crucial role in the development of athletes’ confidence and security, as well as in their ability to cope with difficult situations [39], and social identity has effects on negative emotions [40]. Furthermore, membership in multiple social groups can be a valuable resource for athletes, especially during major transitions in their careers, improving their health and performance [41]. Sports group membership not only affects emotions but also cognitive and physical performance. Athletes who strongly identify with their group tend to perform better due to the cohesion and social support they experience [41,42,43]. Furthermore, continued group membership before and after major transitions in an athlete’s career can have a positive impact on their health and performance.

The perception of the importance of this factor has increased among CLM swimmers, partly due to experiences and activities related to the Aula itself. Actions aimed at promoting long-term work, personal security, and stability by reconciling sport and studies, as well as a higher-level performance environment, appear to be factors highly valued by athletes. In addition, feelings of group and belonging have been fostered among the members, which can imply greater emotional identification and satisfaction. On the other hand, despite raising awareness of other personal entities, it is unclear to what extent they have been involved. We consider it likely that this improvement would have been greater if the activities could have been extended to other environmental elements, such as other sports and educational opportunities.

### 4.3. Athlete’s Family

Recognizing the key role of the family environment, the “Families of Swimmers” program was created to support positive athletic development. Regarding the role of the family, the analysis included parental involvement, emotional support, and personal and financial sacrifice for their children’s careers. This also reflects the influence of parents on different aspects of their children’s values and self-esteem. The perception of the role family plays improved significantly. It is possible that the improvement related to parents promoting positive aspects of motivation, self, and sporting values. Important aspects such as the economic and personal sacrifices of the family, which were not addressed in the program, could be perceived by the swimmers as genuine family sacrifices.

Conversely, the program aimed at parents had positive consequences on their perception of “self” and in the teaching of sporting values. For example, at the level of motivation, there are several studies that show how families can influence motivational orientation and self-determination. Relations between parental motives and child involvement in sport as a function of supportive parenting are likely important and signpost potential targets for family-based behavior change interventions [44,45]. Athletes prefer parents to show interest by facilitating and prioritizing their participation, listening and learning from them, and supporting them before, during, and after sporting events, and they could object to parents providing feedback focused on a demand for results [14,46]. Young people value support in terms of health and social development and the fostering of self-determined motivations. In contrast, they consider parental intervention in aspects of sport and relationships with teammates and opponents undesirable. Excessive parental interference can negatively affect the development of children’s identity and autonomy affecting self-concept, self-esteem, and self-confidence [15,16,17]. Therefore, the results of this four-year program are in line with the scientific literature on the role of the family in the aspects evaluated.

### 4.4. Personal Characteristics

The only area that did not show significant improvements was, rather surprisingly, personal characteristics. The goal was to assess the importance athletes placed on a series of personal characteristics deemed important for athletic success in previous studies [2,12,13]. Swimmers in the CLM team perceived themselves as having greater skill in the emotional, psychological, physical, and cognitive aspects of sport, but these aspects were already valued in this way in 2021. Intervention programs targeted self-confidence, motivation, commitment, attention regulation, arousal regulation, planning, evaluation, emotional regulation, stress management, resilience, mental toughness, coping, goal setting, imagery, and self-talk based on earlier recommendations [2], and promoting these characteristics should promote elite athletes’ performance and well-being [12,13]. The program has most likely improved these self-perceptions, although they are not significant.

It would be easy to attribute the lack of improvement in personal characteristics to shortcomings in the intervention program. Analyzing the existing perceptions swimmers had of themselves before their inclusion, which were already very high, it would not be unreasonable to assert that the lack of improvement was influenced by high personal characteristics related to sports performance. The main criterion for inclusion in this program was belonging to a regional team as a direct consequence of the swimmer’s sporting merits. This inclusion criteria could explain a ceiling effect in the measurement of changes in personal characteristics over the course of the program.

### 4.5. Multi-Area Approach

Another notable aspect of the program is that it has elicited improvements in several of the areas factor studied. This is especially relevant because various studies show each of the areas analyzed can affect one or more of the other areas, for example, where the coach and the family can be prominent in the environment. Nowadays, coaches’ influence can range beyond the athlete, encompassing their environment, through “digital communications” [47]. Digital tools can be used to adopt approaches related to ecological dynamics, aligning with existing research in coaching science and contemporary theories on skill acquisition and motor learning. Numerous studies show the concern shown by coaches in all the elements that surround an athlete, influencing them in the best possible way so that athletes enjoy the best conditions. Coaches who prioritize the caregiving environment are often perceived as key adult figures in the lives of young athletes [20,32,48]. Parents will look for the best environmental conditions and the best coaches for their children. In many cases, it is the parents themselves who, with respect to the opinions of their children, make the decision to choose which club or coach is the most convenient for their children, as they consider it to be the best thing for them [49,50]. Thus, it is clearly important that such a program tries to positively influence several areas of athlete development simultaneously. To our knowledge, studies that not attempt to develop all areas simultaneously have yielded inconclusive results [32,51], and that is why the approach followed in the present study, with simultaneous interventions, was more effective.

Our findings have several practical implications. First, the demonstrated effectiveness of the psychosocial intervention program suggests its potential for implementation by other sports federations and related institutions. This program has shown that it is important for sports entities to influence different areas and not only focus on specific areas. Another relevant aspect is that it emphasizes long-term work through longitudinal programs. The results underscore the importance of designing multifactorial interventions that address the broad range of factors athletes perceive as relevant to their performance, rather than focusing solely on isolated components. Secondly, at the theoretical level, the results emphasize the importance of multifactorial approaches when analyzing the psychosocial development of an athlete.

However, this study was subject to certain limitations; notably, the small sample size, the single-group methodology, and the specificity of the cohort studied constrain the generalizability of the findings to other sports and settings. The absence of a randomized control group was another limitation, as was the loss of subjects, although this issue was controlled for by statistical analysis. Moreover, potential biases may have arisen from self-report measures and social desirability.

Future research should design and implement multi-component psychosocial pro-grams evaluated under a mixed-methods approach to complement quantitative results with athlete and coach narratives. The use of objective measures of behavior and performance is recommended, as well as implementing similar interventions in random or quasi-experimental formats. It would be important to analyze other cohorts of swimmers and athletes from other sports.

## 5. Conclusions

The Aula UCLM-FNCLM program has substantially contributed to improving the perception that athletes have of the role played by their coach in the achievement of their sporting successes, showing a high-power effect. The program improved the perception that athletes have of the role played by their environment and their family in contributing towards their sporting successes with a moderate-power effect. However, the perceptions that athletes have about their personal characteristics in terms of improving their sports performance were largely unchanged. A multi-component support program addressing the coach, family, and environmental and personal characteristics can be beneficial for young, emerging athletes.

## Figures and Tables

**Table 1:** Changes in psychosocial domains after four years of interventions programs for the 2025 Castilla-La Mancha team and swimmers compared with the 2021 Castilla-La Mancha team.

Self-Perception Factors	*Mean*	*SD*	*p*	*η* ^2^	(1 − *β*)	95% C I Limits	
						Lower	Upper
Coach CLM 2025 Coach CLM 2021	8.5	2.2	<0.001 *	0.30	0.98	7.79	9.21
	4.1	0.9				3.80	4.37
Environment CLM 2025 Environment CLM 2021	7.6	2.0	<0.05 ***	0.11	0.58	6.95	8.23
	3.7	1.7				3.20	4.33
Personal CLM 2025 Personal CLM 2021	8.2	2.0	n.s.			7.58	8.84
	6.9	1.4				6.53	7.45
Family CLM 2025 Family CLM 2021	7.9	2.2	<0.05 ***	0.12	0.60	7.22	8.61
	5.1	2.5				4.36	5.94

Note: Mean = mean Likert scale value (CAED for each factor); SD = standard deviation. * (*p* < 0.001), and *** (*p* < 0.05). *η*^2^ = effect Size. Partial eta squared (1 − *β*) = effect power; n.s. = non-significant differences.

## Data Availability

Data are available on request.

## Abbreviations

The following abbreviations are used in this manuscript:

CLM	Castilla-La Mancha
CAED	Questionnaire on self-perception of performance in sport
AULA	Aula UCLM-FNCLM to promote swimming and health
